# Supplementary material for: Environmental permittivity-asymmetric BIC metasurfaces with electrical reconfigurability
Source: Nat Commun. 2024 Aug 15;15:7050. doi: 10.1038/s41467-024-51340-7 (PMC11327280; doi:10.1038/s41467-024-51340-7)
Supplement: Supplementary file 1 — Supplementary Information [file 41467_2024_51340_MOESM1_ESM.pdf]

Supplementary Information for

Environmental permittivity-asymmetric BIC metasurfaces with electrical reconfigurability

Haiyang Hu[1,4], Wenzheng Lu[1,4], Alexander Antonov[1], Rodrigo Berté[1], Stefan A Maier[2,3], Andreas Tittl[1]*

1. Chair in Hybrid Nanosystems, Nanoinstitute Munich, Faculty of Physics, Ludwig-Maximilians-Universität München, Königinstraße 10, 80539 München, Germany.

2. School of Physics and Astronomy, Monash University Clayton Campus, Melbourne, Victoria 3800, Australia.

3. The Blackett Laboratory, Department of Physics, Imperial College London, London SW7 2AZ, United Kingdom.

4. These authors contributed equally

*E-mail: Andreas.Tittl@physik.uni-muenchen.de

Tables of contents

Supplementary Note 1. Multipole analysis of permittivity-asymmetric BIC-metasurface.

Supplementary Figure 1. Multipole analysis of asymmetric permittivity in *q*BIC metasurfaces.

Supplementary Figure 2. Schematic overview of nanofabrication for PMMA based environmental permittivity-asymmetric BIC metasurfaces.

Supplementary Figure 3. Numerical simulation of the ε-*q*BICs metasurfaces with varied n_1.

Supplementary Figure 4. Qualify factor analysis of simulated and experimental PMMA based ε-*q*BICs metasurfaces.

Supplementary Figure 5. The shifting of qBIC resonances during the PANI growing and switching processes.

Supplementary Figure 6. Numerical simulation of *q*BIC resonances with varied refractive index of surrounding medium on geometry-asymmetric unit cells.

Supplementary Figure 7. Geometry induced symmetry breaking of *q*BIC metasurfaces.

Supplementary Figure 8. *q*BIC resonances based on metasurfaces with geometric asymmetry and permittivity asymmetry.

Supplementary Figure 9. ε-*q*BIC metasurfaces with varied scaling factors.

Supplementary Figure 10. Numerical simulation of ε-*q*BIC metasurfaces with varied h_{PMMA}.

Supplementary Figure 11. Simulated electric field distribution of the unit cell and the regional integration analysis.

Supplementary Figure 12. Simulated transmittance spectra of ε-*q*BICs metasurfaces using various photoresists.

Supplementary Figure 13. Measured refractive index and extinction coefficient of PANI in different states.

Supplementary Figure 14. Analysis of PANI thickness during the in-situ coating process.

Supplementary Note 1. Multipole analysis of permittivity-asymmetric BIC-metasurface

Let's start with a non-symmetry-breaking scenario, where two identical dielectric rods host antiparallel-dipole resonance collinear with the y-axis (Supplementary Figure 1a). In the absence of PMMA cladding, the system exhibits symmetry-protected BIC with equal antiparallel dipole moments: $\mathbf{p}_1 = -\mathbf{p}_2$. Such a system possesses two mirror planes symmetry which intersection forms rotational vertical axis C_2. The coupling coefficient of the eigenstate with the normally incident light along the z-direction with a wave vector $k = \omega/c$ and linear polarization along $\mathbf{e}_y$ is proportional to an overlap integral:[1]

$$m_y \propto \int_{V_1,V_2} \mathbf{J}(\mathbf{r}) \cdot \mathbf{e}_y \, e^{i\mathbf{k}\cdot\mathbf{r}} dV \propto (\mathbf{p}_1 + \mathbf{p}_2) \cdot \mathbf{e}_y = 0, \quad (1)$$

where $\mathbf{J}(\mathbf{r})$ is a displacement current density, V_1 and V_2 are the volumes of the dielectric nanorods.

Covering one of the nanorods with PMMA clearly breaks one of the mirror plane symmetries and transforms BIC into radiative linearly polarized *quasi*-BIC (*q*BIC). To reveal more insight into how exactly PMMA cladding affects resonance, we perform multipole expansion of the coupling coefficient:

$$m_e \propto \int_V \mathbf{J}(\mathbf{r}) \cdot \mathbf{e} \, e^{i\mathbf{k}\cdot\mathbf{r}} dV \approx \int_V \mathbf{J}(\mathbf{r}) \cdot \mathbf{e} \, (1 + i\mathbf{k}\cdot\mathbf{r}) dV$$

$$\approx -i\omega \mathbf{P} \cdot \mathbf{e} + i(\mathbf{k} \times \mathbf{M}) \cdot \mathbf{e} - \frac{\omega}{6} (\mathbf{e})_\alpha (\mathbf{k})_\beta Q_{\alpha\beta}, \quad (2)$$

where the dipole, magnetic, and quadrupole electric moments are introduced in a standard way:[2]

$$\mathbf{P} = \frac{i}{\omega} \int_V \mathbf{J}(\mathbf{r}) \, dV, \quad (3)$$

$$\mathbf{M} = \frac{1}{2} \int_V \mathbf{r} \times \mathbf{J}(\mathbf{r}) \, dV, \quad (4)$$

$$Q_{\alpha\beta} = \frac{3i}{\omega} \int_V \left[r_\alpha J_\beta(\mathbf{r}) + r_\beta J_\alpha(\mathbf{r}) - \frac{2}{3} \delta_{\alpha\beta} \mathbf{r} \cdot \mathbf{J}(\mathbf{r}) \right] dV, \quad (5)$$

Taking into account incident wave polarization one can easily reduce Equation (2):

$$m_y \propto P_y - \frac{1}{c} M_x - \frac{i\omega}{6c} Q_{yz}, \quad (6)$$

Next, we employ numerical simulation by COMSOL Multiphysics to compare the contributions of all terms to the coupling coefficient. We set geometrical sizes according to Figure 2 of the main text. For simplicity, we also removed the ITO layer and set constant refractive indexes: $n_{\mathrm{TiO_2}} = 2.5$ and $n_{\mathrm{SiO_2}} = 1.45$. We solve the problem by using the Electromagnetic Waves, Frequency Domain module, and Wavelength Domain study for one unit cell with the periodic boundary conditions on the sides. A periodic port located at the top of the modeling domain generates light with the corresponding linear polarization. The calculated transmittance spectrum, featuring the pronounced qBIC at the wavelength $\lambda \approx 688$ nm is given in Supplementary Figure 1c.

For the multipole analysis, we integrate the calculated fields separately over both nanorods and PMMA according to the Equations (3)-(5). Supplementary Figure 1c shows the contributions of all terms of the Equation (6). As expected, the main contribution comes from nanorods due to electric dipole moments P_y, which have almost the same shapes and opposite signs within each rod. While, the role of the magnetic dipole M_x and electric quadrupole Q_{yz} moments cannot be neglected: their corresponding absolute value at quasi-BIC resonance is only 3 times less than the value of P_y. Regarding the PMMA coverage, its predominant contribution arises from electric dipole moments P_y. Although its peak value is almost 5 times less than that of rods, it is enough to transform non-radiative BIC into a well-pronounced qBIC.

Supplementary Figures

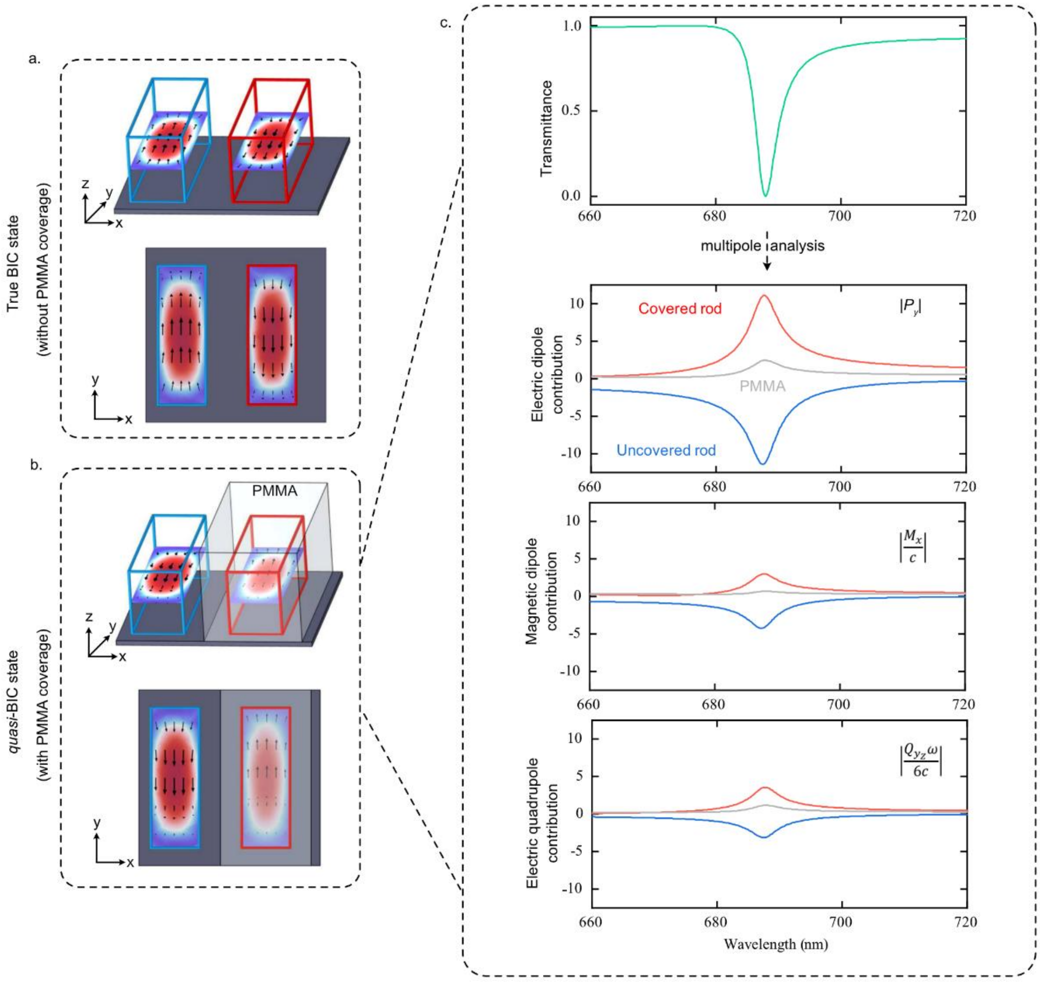

Supplementary Figure 1. Multipole analysis of permittivity-asymmetric BIC metasurfaces. a. Simulated electric field distribution in arb.u. over the middle cut of the nanorods at the perfect BIC (without PMMA coverage) and b. qBIC (with PMMA coverage) resonant wavelengths. c. Transmittance spectra of qBIC metasurfaces, and contributions of electric dipole P_y, magnetic dipole M_x and electric quadrupole Q_{yz} moments in arb.u. provided by the rod immersed in the PMMA (red lines), uncovered rod (blue lines), and PMMA itself (gray lines).

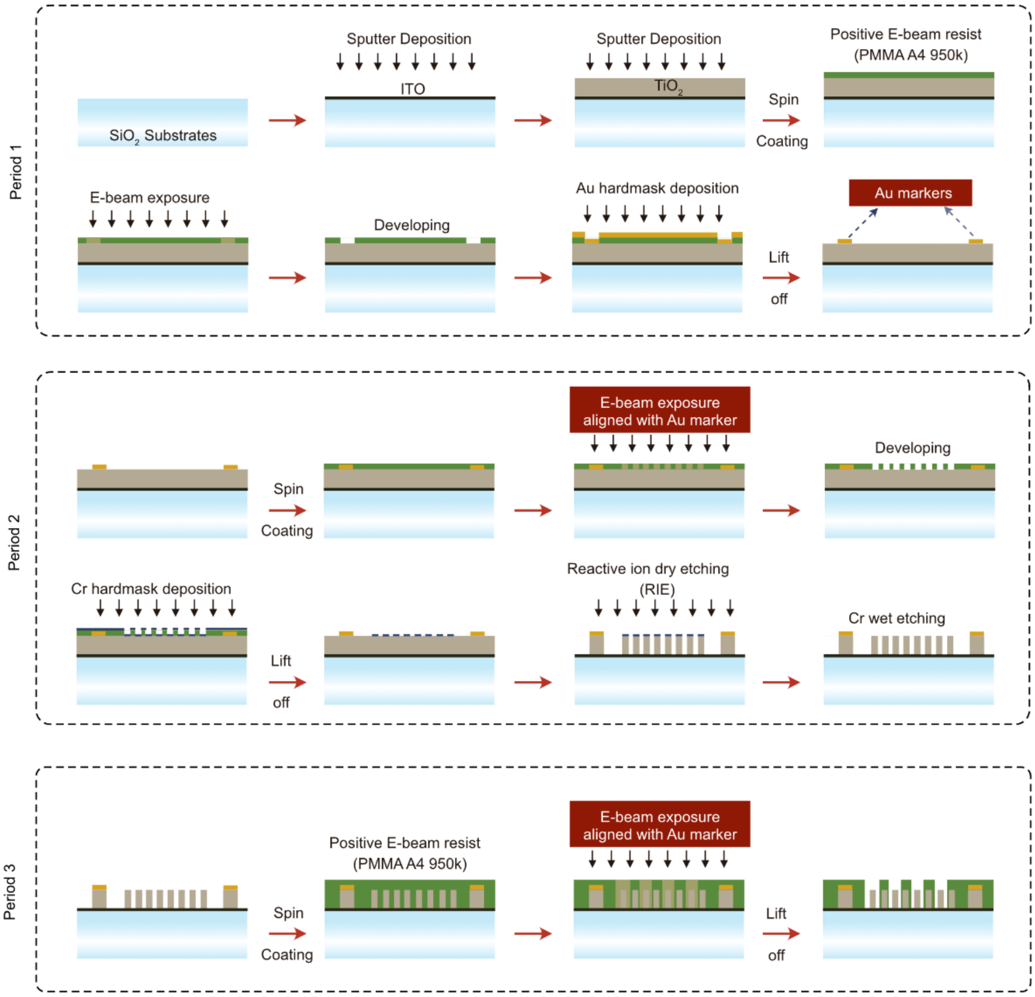

Supplementary Figure 2. Schematic overview of nanofabrication for PMMA based environmental permittivity-asymmetric BIC metasurfaces.

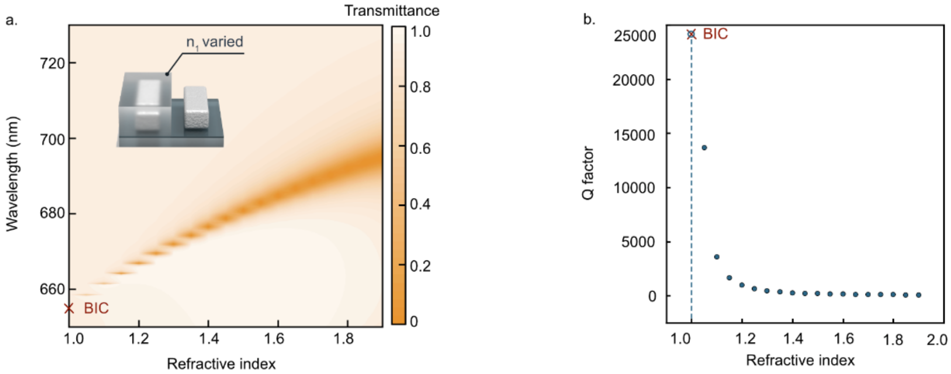

Supplementary Figure 3. Numerical simulation of the ε-qBICs metasurfaces with varied n_1. a. Color-coded simulated transmittance map of the ε-qBICs metasurfaces as

a function of the wavelength and the refractive index of the surrounding medium on one row (n_1). Insert schematic shows the unit cell of the ε-*q*BICs metasurface, where n_1 is variable while n_2 = 1. b. The quality factor (Q factor), of the ε-*q*BIC resonance extracted from the transmittance spectra.

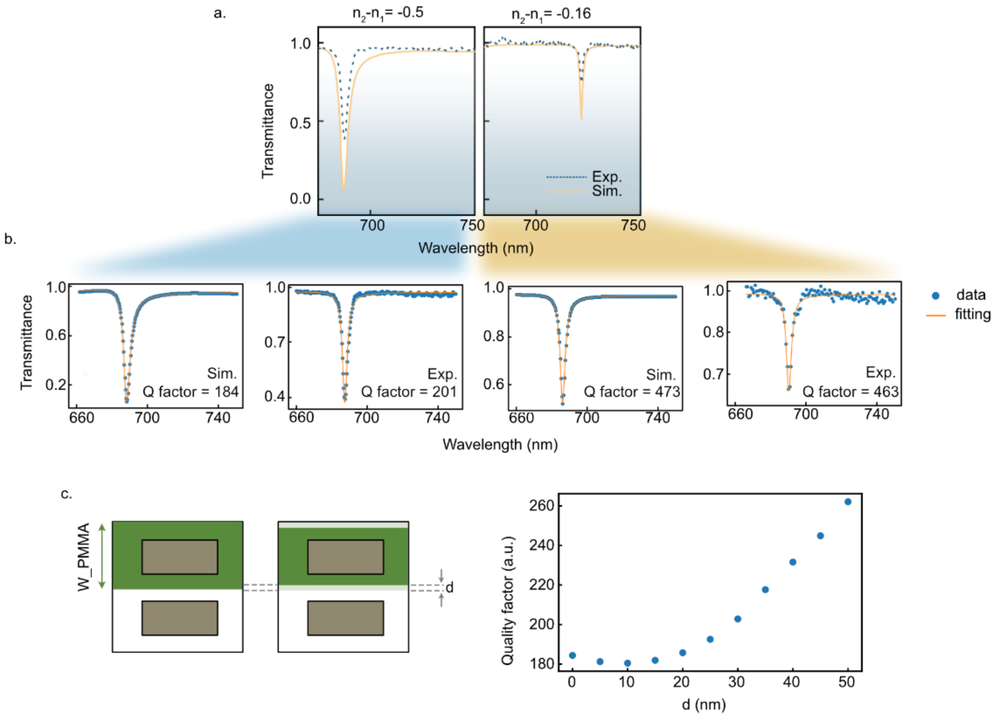

Supplementary Figure 4. Qualify factor analysis of simulated and experimental PMMA based ε-*q*BICs metasurfaces. a. Experimental and numerical transmittance spectra confirm the high reconfigurability of ε-*q*BICs metasurfaces through customized refilling of different environmental media (air, water) for tuning the refractive index contrast (Δn) surrounding media of the two rods (appending to Figure 2). b. Quality factors are extracted based on the fitting with temporal mode coupling theory. c. The variance of quality factor in experimental fabrication compared to simulations is due to the slightly smaller coverage ratio of PMMA. The width of PMMA coverage on the unit cell was designed as half of the unit cell, which could be slightly different during the experiment because of the fabrication tolerances.

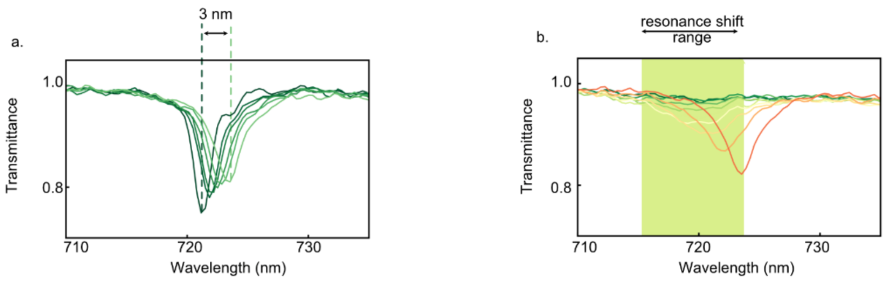

Supplementary Figure 5. The shifting of qBIC resonances during the PANI growing and switching processes. a. Experimental in-situ transmittance measurements of ε-qBIC metasurfaces with different PANI coating cycles (0, 36, 42, 48, 54, 60) in the reduced state. The transmittance shift of approximately 3 nm is indicated (color map corresponds to Figure 3c). b. Experimental transmittance spectra at various applied voltages, demonstrating the transition of ε-qBICs between high (ON) and low (OFF) transmittance states. The resonance shift range is highlighted (the color map corresponds to Figure 4b).

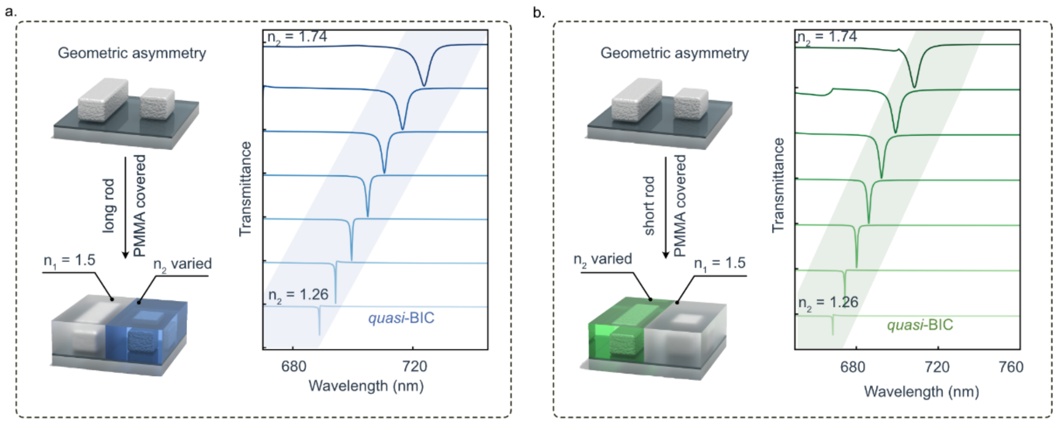

Supplementary Figure 6. Numerical simulation of *q*BIC resonances with varied refractive index of surrounding medium on geometry-asymmetric unit cells. a. Schematics of the unit cell by covering the long rod with PMMA, with the shorter rod exposed to a medium of varying refractive index (left). Alongside are the corresponding to the simulated transmittance spectra (right). b. Schematics of the unit cell by covering the short rod with PMMA (left), and their corresponding simulated transmittance spectra (right).

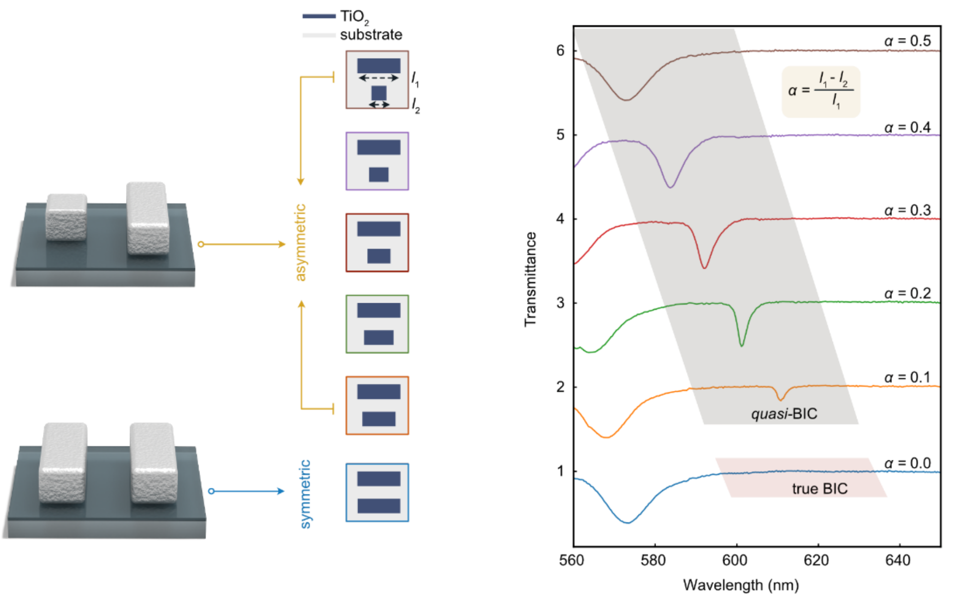

Supplementary Figure 7. Geometry induced symmetry breaking of *q*BIC metasurfaces. The geometry asymmetry in the TiO_2 two-rod unit cell is realized by shortening the length of one of the rod, as shown in the schematics (left). The geometric asymmetry α, is defined by $\alpha = \frac{l_1 - l_2}{l_1}$, where l_1 and l_2 are the length of the longer rod and shorter rod, respectively. The experimentally measured transmittance spectra of the *q*BIC metasurfaces with different α are shown (right). When the unit cell is geometrically symmetry, the BIC is symmetry-protected and no BIC resonance is displayed in the spectrum. The *q*BIC emerges with the presence of geometric asymmetry. When the geometric asymmetry α is increased, the *q*BIC blueshifts and broadens in the peak width.

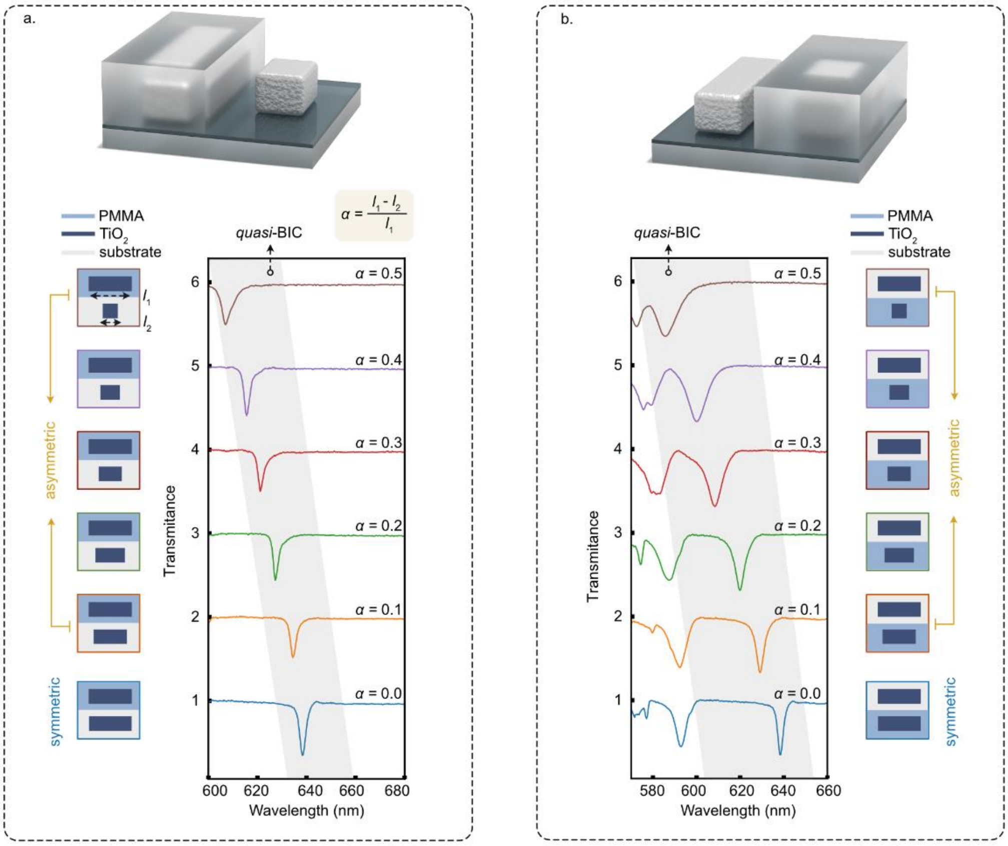

Supplementary Figure 8. *q*BIC resonances based on metasurfaces with geometric asymmetry and permittivity asymmetry. a. *q*BIC metasurfaces with PMMA covered on the long rod, while the short rod is exposed to air. The geometric asymmetry α, is defined by the ratio of the length difference of the two rods to the length of the long rod, as shown by the equation in the figure. b. *q*BIC metasurfaces with PMMA covered on the short rod, while the long rod is exposed to air. For both cases, as the geometric asymmetry increases, the *q*BIC blueshifts.

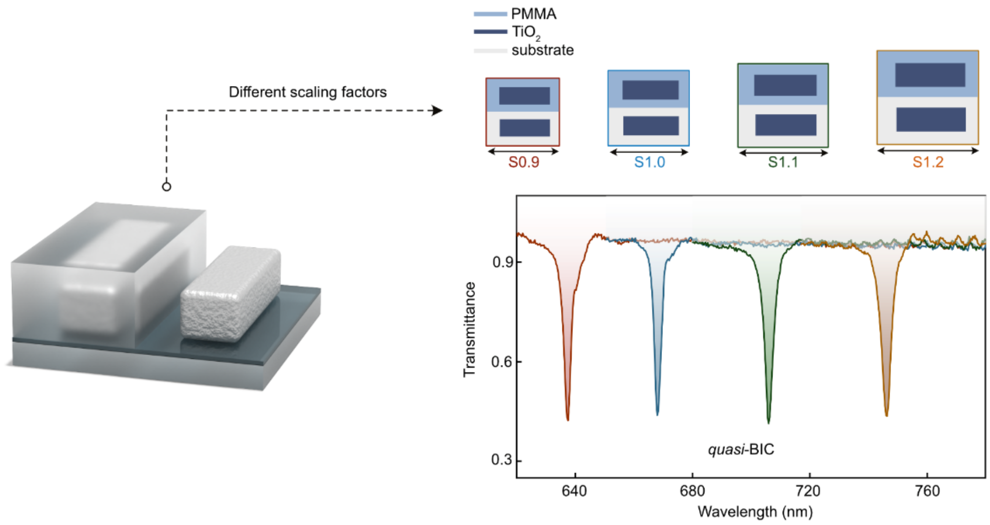

Supplementary Figure 9. Experimental transmittance spectra of ε-*q*BIC metasurfaces with varied scaling factors. Schematics (left) and transmittance spectra (right) of the ε-*q*BIC metasurfaces with varied scaling factors from 0.9 to 1.2. As the scaling factor is increased, the corresponding generated ε-*q*BIC redshifts.

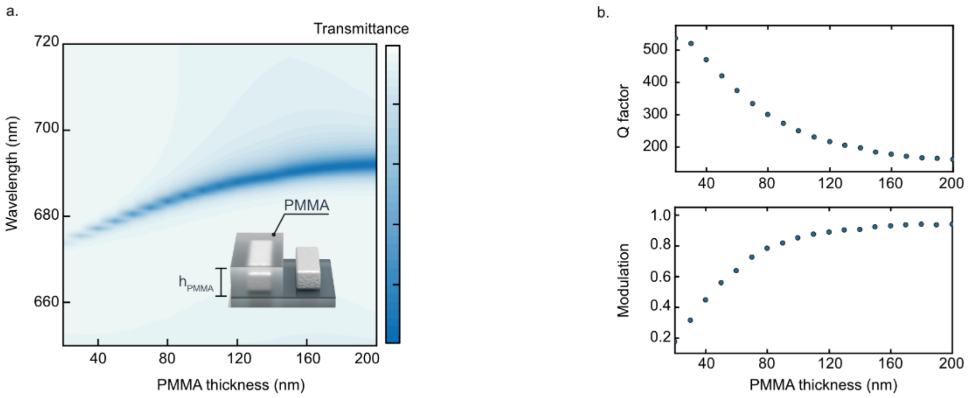

Supplementary Figure 10. Numerical simulation of ε-*q*BIC metasurfaces with varied h_{PMMA}. a. Color-coded maps for transmittance spectra of ε-*q*BICs metasurfaces as a function of wavelength and PMMA thickness (h_{PMMA}). Inserted schematic shows the unit cell of the ε-*q*BIC metasurfaces, where h_{PMMA} is varied. The PMMA covers on one rod in the unit cell, while the other rod is exposed to air. b. Analysis of the *Q* factor (top) and the modulation (bottom) of the *q*BICs resonances, derived from transmittance spectra across PMMA coating thicknesses, presented as upward and downward trends.

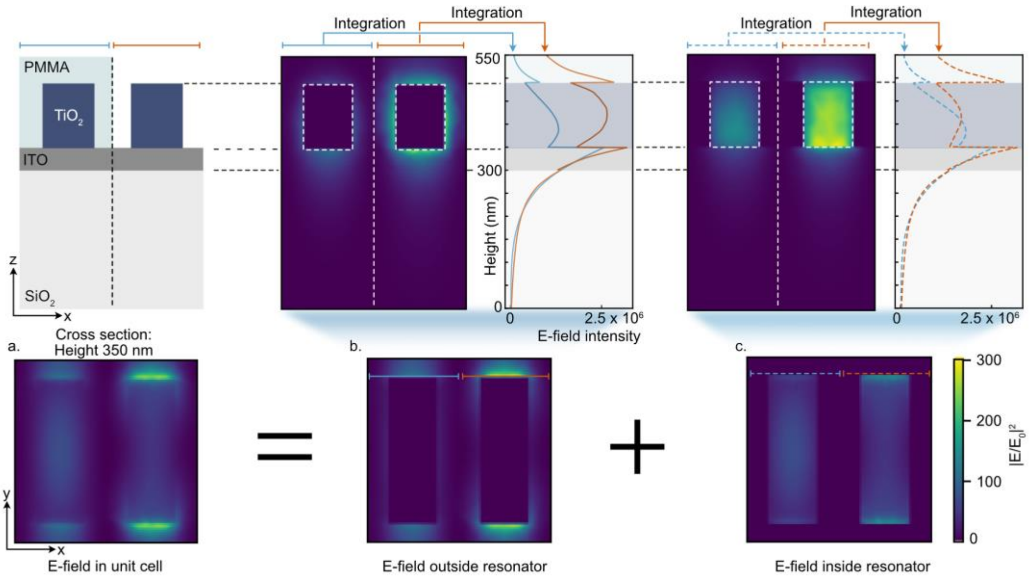

Supplementary Figure 11. Simulated electric field distribution of the unit cell and the regional integration analysis. a. The E-field distribution of the unit cell at the bottom of the TiO_2 resonators, where the left TiO_2 rod resonator is embedded with PMMA and the right TiO_2 rod resonator is exposed to air. The E-field in the unit cell can be divided into two parts, including b. the E-field outside the TiO_2 two-rod resonators, and c. the E-field inside the TiO_2 two-rod resonators. The top figures show the integrated E-field distribution profiles along the *z*-axis direction. The e-field intensities of both the outside and inside of the uncovered TiO_2 rod are generally higher than the one covered by PMMA. The asymmetry of the e-field distribution gives rise to radiation coupling to the far field, exhibiting strong ε-*q*BIC resonances.

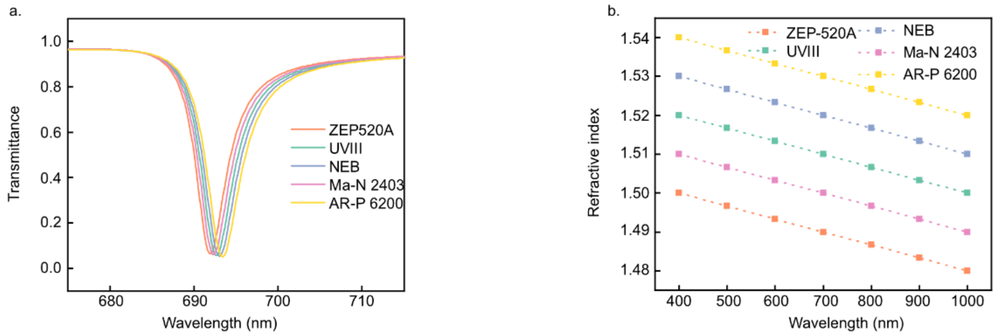

Supplementary Figure 12. Simulated transmittance spectra of ε-*q*BICs metasurfaces using different photoresists. a. Simulated transmittance spectra of the ε-*q*BICs metasurfaces, where one rod in the unit cell is covered by different photoresists with

the thickness of 200 nm. b. The refractive indices of the e-beam photoresists with linear interpolation used in the simulation.

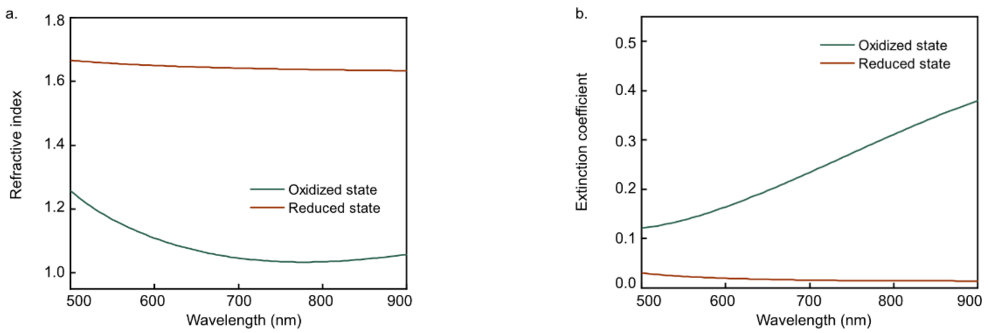

Supplementary Figure 13. Measured refractive index and extinction coefficient of PANI in different states. a. The refractive index of PANI in oxidized and reduced states. b. The extinction coefficient of PANI in oxidized and reduced states.

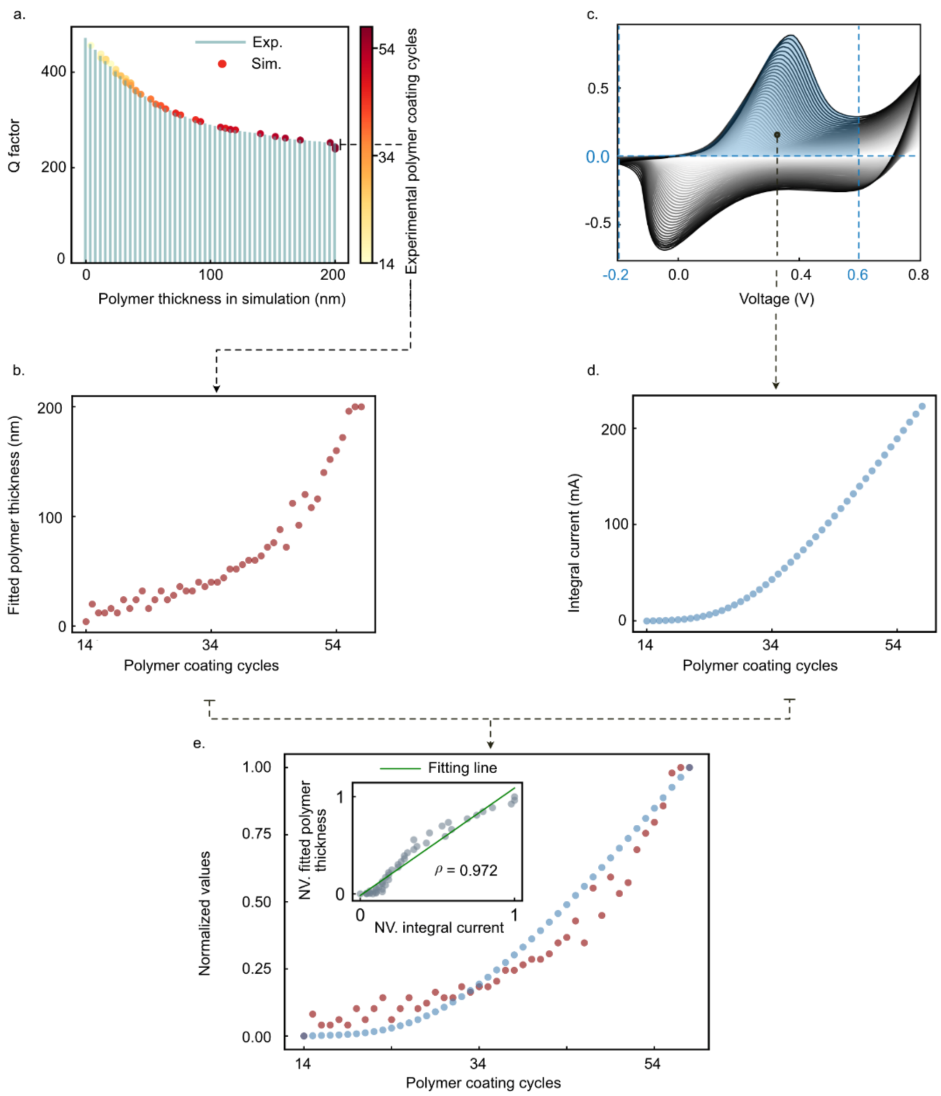

Supplementary Figure 14. Analysis of PANI thickness during the in-situ coating process. a. *Q* factor analysis for the PANI thickness in the simulation and the PANI coating cycle in the experiment. The initial 13 PANI coating cycles are used for system stabilization, during which no observable PANI growth signal is detected. Therefore, our analysis begins from the 14th cycle. The *Q* factors obtained from the simulated and experimental results show excellent agreement. Thus, the *Q* factors can be used to evaluate the PANI thickness during the in-situ coating process. b. The fitted PANI thickness for different coating cycles. The polymer thickness fitting is based on the *Q* factors obtained in the simulation. c. Electrochemical current-voltage diagram recorded during the in-situ PANI coating process. d. Electrochemical integral current for different coating cycles during the in-situ coating process. The integral current is obtained in the range of applied voltage from -0.2 V to +0.6 V in the oxidation

process for each polymer coating cycle, reflecting the total amount of PANI coated on the metasurfaces. e. Comparison of the fitted PANI thickness and the integral current at different coating cycles. Inserted figure shows the analysis of the discrepancy between the *Q*-factor-based fitted PANI thickness and the integral current. The fitted polymer thickness obtained from the *q*BIC resonance *Q* factors shows a similar growing trend to the total amount of coated polymer on the substrate. The polymer thickness analyses from both optical BIC resonance and electrical measurement are in good agreement, strengthening the reliability of the PANI thickness evaluation during the in-situ coating process. Therefore, through monitoring the *Q* factor of the ε-*q*BICs, it further provides insights of the variation of the surrounding media of the resonators.

Supplementary References

1. K. Koshelev, S. Lepeshov, M. Liu, A. Bogdanov, and Y. Kivshar. Asymmetric Metasurfaces with High-Q Resonanaces Governed by Bound States in the Continuum. Phys. Rev. Lett. 121, 193903 (2018).
2. A. Evlyukhin, T. Fischer, C. Reinhardt, and B. Chichkov, Optical theorem and multipole scattering of light by arbitrarily shaped nanoparticles, Phys. Rev. B, 94, 205434 (2016).
